# TTTCA Repeat Expansion of *SAMD12* in a New Benign Adult Familial Myoclonic Epilepsy Pedigree

**DOI:** 10.3389/fneur.2020.00068

**Published:** 2020-02-26

**Authors:** Chaorong Liu, Yanmin Song, Ying Yuan, Ying Peng, Nan Pang, Ranhui Duan, Wen Huang, Xuehui Qin, Wenbiao Xiao, Hongyu Long, Sha Huang, Pinting Zhou, Lili Long, Bo Xiao

**Affiliations:** ^1^Department of Neurology, Xiangya Hospital, Central South University, Changsha, China; ^2^Department of Emergency, Xiangya Hospital, Central South University, Changsha, China; ^3^Department of Endocrinology and Metabolism, The Second Xiangya Hospital, Central South University, Changsha, China; ^4^NHC Key Laboratory of Birth Defects Research, Prevention and Treatment, Hunan Provincial Maternal and Child Health Care Hospital, Changsha, China; ^5^Department of Pediatric, Xiangya Hospital, Central South University, Changsha, China; ^6^Center for Medical Genetics and Hunan Key Laboratory of Medical Genetics, School of Life Sciences, Central South University, Changsha, China; ^7^Department of Neurology, First Hospital of Qinhuangdao, Qinhuangdao, China

**Keywords:** benign adult familial myoclonic epilepsy, *SAMD12*, pentanucleotide, cognitive impairment, TTTCA expansion

## Abstract

Benign adult familial myoclonic epilepsy (BAFME) is an autosomal dominant disorder characterized by adult-onset cortical myoclonus with or without seizures. Recently, it was reported to be associated with intronic TTTTA/TTTCA expansions. To investigate whether these abnormal expansions are involved in our new pedigree from China, whole exome sequencing (WES) and repeat-primed polymerase chain reaction (RP-PCR) analysis were performed to detect potential mutation in pedigree members. Neither causal mutations cosegregated with the disease in the family nor any novel mutation was identified through WES, while an abnormal TTTCA expansion in *SAMD12* was identified by RP-PCR and then proved to be cosegregated in the pedigree. All the 12 alive affected individuals (M/F = 4/8; average age = 46.7 years old, range from 27 to 66) showed typical characteristics of BAFME. In addition, maternal clinical anticipation was observed in six mother/child pairs. In conclusion, our study offered the evidence of intronic pentanucleotide expansions in *SAMD12* from a new Chinese BAFME pedigree, which further confirmed the association between this expansion and the pathogenesis of BAFME.

## Introduction

Benign adult familial myoclonic epilepsy (BAFME), also known as familial cortical myoclonic tremor with epilepsy (FCMTE) and autosomal dominant cortical myoclonus and epilepsy (ADCME), was first reported in the 1990's in Japan (1, 2). It is an autosomal dominant disease featured by adult-onset cortical myoclonus with or without seizures and developing with a benign course. Giant somatosensory-evoked potential and long-latency cortical reflex can be detected by electromyography in patients with BAFME, which collectively support the cortical origin of the cortical tremor (3). Seven subtypes have been reported so far, including BAFME1 (8q24, OMIM 601068) (4), BAFME 2 (2p11.1–q12.2, OMIM 607876) (5), BAFME 3 (5p15.31–p15.1, OMIM 613608) (6), BAFME 4 (3q26.32–3q28, OMIM 615127) (7), BAFME 5 (1q32.1, OMIM 615400) (8), BAFME 6 (16p12.1, OMOM 618074) (4), and BAFME 7 (4q32.1, OMIM 618075) (4). Thus, BAFME showed genetic heterogeneity among different families. Recently, pentanucleotide expansions of intronic TTTCA and TTTTA were found to be involved in the pathogenesis of BAFME 1 in Japanese pedigrees regardless of the gene location (4). Three more recent reports also confirmed TTTCA pentanucleotide expansions in BAFME 1 families of another ethic background (9–11). Subsequently, TTTCA repeat insertions in an intron of *YEATS2* in BAFME 4 was confirmed in a Thailand pedigree (7). ATTTC repeat expansions in *STARD7* and unstable TTTTA/TTTCA expansions in *MARCH6* were also found in European families (5, 6). Here, we performed mutation analysis in a new BAFME family from central China and detected whether abnormal insertion of TTTTA and TTTCA expansions were associated with this BAFME pedigree.

## Participants and Methods

### Participants

**Figure 1:**
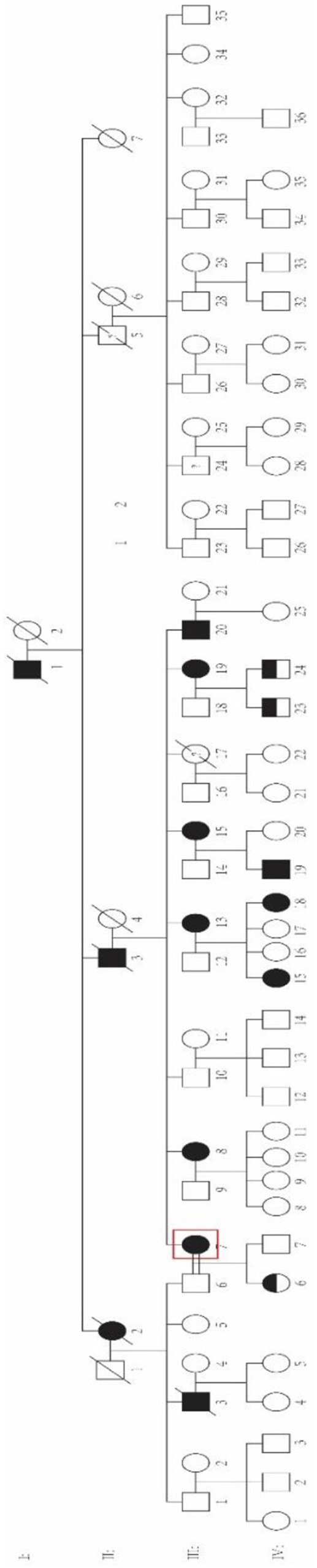
Pedigree of the family. The red box represents the proband.

The family was a four-generation Chinese pedigree with 80 members, which presents as autosomal dominant inheritance (Figure 1). All available individuals underwent detailed medical history collection and physical examination by two experienced neurologists. Brain MRI, electroencephalogram (EEG), and electromyogram (EMG) examinations were collected at the same time; Unified Myoclonus Rating Scale (UMRS) and Fahn Tremor Rating Scale (FTRS) were assessed to evaluate the severity of myoclonus and tremor, respectively. Mini-Mental State Examination (MMSE) and Digit Symbol Test (DST) were used to evaluate the cognitive function. Ten gender- and age-matched unrelated healthy controls were recruited to detect whether there were cognitive dysfunction in BAFME patients. Genomic DNA was extracted from peripheral venous blood using the Genomic DNA kit from Aidlab. This study was approved by the Ethics Committee of Xiangya Hospital, Central South University. Written informed consent was obtained from all enrolled participants.

### Whole-Exome Sequence Analysis

Five members (IV19, III13, III14, III10, and IV6) from the pedigree were performed whole-exome sequence (WES) analysis using HiSeq2000 (Illumina, PE150) following the manufacturer's protocol. Sequenced reads were aligned to GRCh37/hg19 using Burrows–Wheeler Aligner (BWA) (v.0.7.12) with default parameters. SAMtools (v0.1.12) was used to call the Variants and RefSeq Genomes database; 1000 Genomes database (http://www.1000genomes.org/), and ExAC database (http://exac.broadinstitute.org/) were used to annotate the Variants.

### Repeat-Primed Polymerase Chain Reaction Analysis

Repeat-primed polymerase chain reaction (RP-PCR) analysis targeting TTTTA and TTTCA expansions in *SAMD12* was performed using the primers (listed in Supplementary Table 1) with ABI3730xl DNA Analyzer. The reaction mixture (10 μl) included 1 × GC buffer I (Takara), 0.3 mM deoxynucleoside triphosphate (dNTP), 0.2 μM of each primer, 0.3 U HotStarTaq polymerase (Takara), and 1 μl template DNA. The cycling program was 95°C, 2 min; 11 cycles × (94°C, 20 s, 56–0.5°C/cycle 40 s, 72°C, 3 min); 25 cycles × (94°C, 20 s; 57°C, 30 s; 72°C, 3 min); 72°C, 10 min; 4°C forever. PCR product was analyzed on the ABI3730XL sequencer. The results were analyzed with Genemapper (v5.0) software and combined with manual proofreading. Every experiment was performed three times (primers used for RP-PCR are listed in Supplementary Table 1).

### Statistics Analysis

Continuous variables are presented as means (± standard deviation), and categorical variables are shown as numbers (%). SPSS version 23.0 for Windows (SPSS Inc., Chicago, IL, United States) was used for calculation. Pearson correlation analysis was conducted to evaluate the relationship between the severity (FTRS, UMRS) and the duration of the disease. Two-sample *t*-test was used to assess the cognitive difference between BAFME patients and unrelated healthy controls. Significance was set at *p* < 0.05.

## Results

### Clinical Details of Affected Individuals

All the 12 alive affected individuals (M/F = 4/8; average age = 46.7 years old, range from 27 to 66) showed typical characteristics of BAFME. Two affected individuals presented only tremor symptoms (IV-23 and IV-24), and one with both tremor and myoclonus (IV-6), while another two members showed seizures only (III-8 and IV-18); the remaining seven patients manifested both tremor and infrequent epilepsy, among which four presented myoclonus together. Among the seven individuals, tremor appeared earlier than epilepsy in four individuals by an average of 4 years (50%), two appeared in the same year (25%), while for the remaining two patients, seizure appeared in advance with 2.5 years (25%). Tremor was present in adults, with an average age of onset of 35.4 ± 8.68 years in all affected members. Myoclonus appeared in the same year with tremor (3/4) or 2 years after it (1/4). Neither the severity of tremor nor myoclonus is correlated to its duration (for FTRS, *r* = 0.42, *p* = 0.196; for UMRS, *r* = −0.93, *p* = 0.071). Seizures were also present in adults, with an average age of onset of 35 ± 5.52 years, and all manifested as generalized tonic–clonic seizure, with a frequency of once to three times a year. Six patients took antiepilepsy drugs phenobarbital, of which all became seizure free.

No significant difference was found in the age between patients and unrelated healthy controls (*p* = 0.079, two-sample *t*-test). There was significant difference between the two groups in MMSE score (28.2 ± 8.03 vs. 29.7 ± 0.46, *p* = 0.005) and Verbal Fluency Test (VFT) (14.3 ± 3.71 vs. 20.1 ± 4.67, *p* = 0.018), whereas no significant difference was found in DST (41.3 ± 24.10 vs. 52.5 ± 24.94, *p* = 0.093). No significant correlation was found between minimum repeat size estimated by RP-PCR and the onset age of BAFME (*r* = −0.477, *p* = 0.163) (Supplementary Table 3).

Clinical anticipation was observed in both symptoms of tremor and seizure, with an average anticipation of 14 and 5 years, respectively (Table 1).

**Table 1:** Summary of parent/child pairs.

**Parent/child pairs**	**Transmission type**	Clinical anticipation^*^ (years)	
		**Cortical tremor**	**Seizure**
III-7/IV-6	Maternal	5	–
III-13/IV-15	Maternal	8	5
III-13/IV-18	Maternal	–	6
III-15/IV-19	Maternal	7	5
III-19/IV-23	Maternal	26	–
III-19/IV-24	Maternal	28	–

* *Years of onset age anticipated; “–”,no anticipation observed; “/”, data unavailable*.

**Figure 2:**
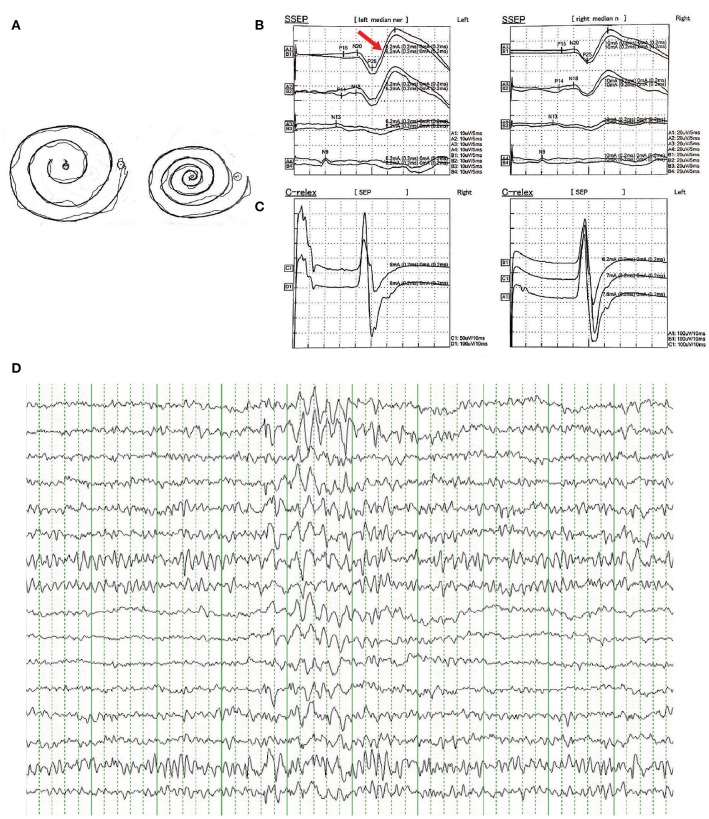
**(A)** Free-hand drawing test of Archimedes' spiral showed mild irregularity in patients of the family. **(B)** Representative result of somatosensory-evoked potential (SEPs) in bilateral median nerve of wrist showed enhanced cortical components (giant potentials and increase in N20-P25 indicated by red arrow). **(C)** Representative result of C-reflex. **(D)** Representative result of epileptiform discharges recorded in EEG showed bilateral bursts with high-amplitude θ-wave, spikes, and incompletely sharp complex waves were recorded mainly in the right frontal region and left posterior temporal region.

There were no other neurological or psychiatric diseases presented in all affected individuals. Drawing test of Archimedes' spiral showed mild to severe irregularity. Brain MRI showed no obvious abnormality. EMG represented a giant somatosensory-evoked potential (SEP) and C-reflex by stimulating the median nerve in bilateral wrist. EEG showed bilateral bursts with high-amplitude θ-wave; spikes and incompletely sharp complex waves were recorded mainly in the right frontal region and left posterior temporal region. These results indicated the cortical origin of the tremors or myoclonus. Detailed clinical information is displayed in Table 2 and Figure 2.

**Table 2:** Clinical details of the family.

**Pedigree ID**	**Gender**	**Age**	**Onset age of**			**Psychomotor development**	**EEG**	**SEPs**	**MRI**	**AEDs**
			**Tremor**	**myoclonus**	**seizure**					
III-7	Female	62	44	N	41	N	+	+	N	phB
III-8	Female	66	N	N	30	N	+	+	N	UT
III-13	Female	60	38	N	33	N	+	+	N	phB
III-15	Female	59	35	N	35	N	+	+	N	PhB
III-19	Female	50	30	30	26	N	+	+	N	phB
III-20	Male	47	32	32	32	N	+	+	N	phB
IV-6	Female	41	39	39	N	N	+	+	N	UT
IV-15	Female	40	30	N	28	N	+	+	N	phB
IV-18	Female	30	N	N	27	N	+	+	N	UT
IV-19	Male	33	28	30	30	N	+	+	N	UT
IV-23	Male	29	28	N	N	N	+	+	N	UT
IV-24	Male	27	25	N	N	N	+	+	N	UT

*phB, phenobarbitone; +, abnormal, N, normal; UT, untreated; AED, anti-epilepsy drug; SEP, somatosensory-evoked potential*.

### Mutation Screening

**Figure 3:**
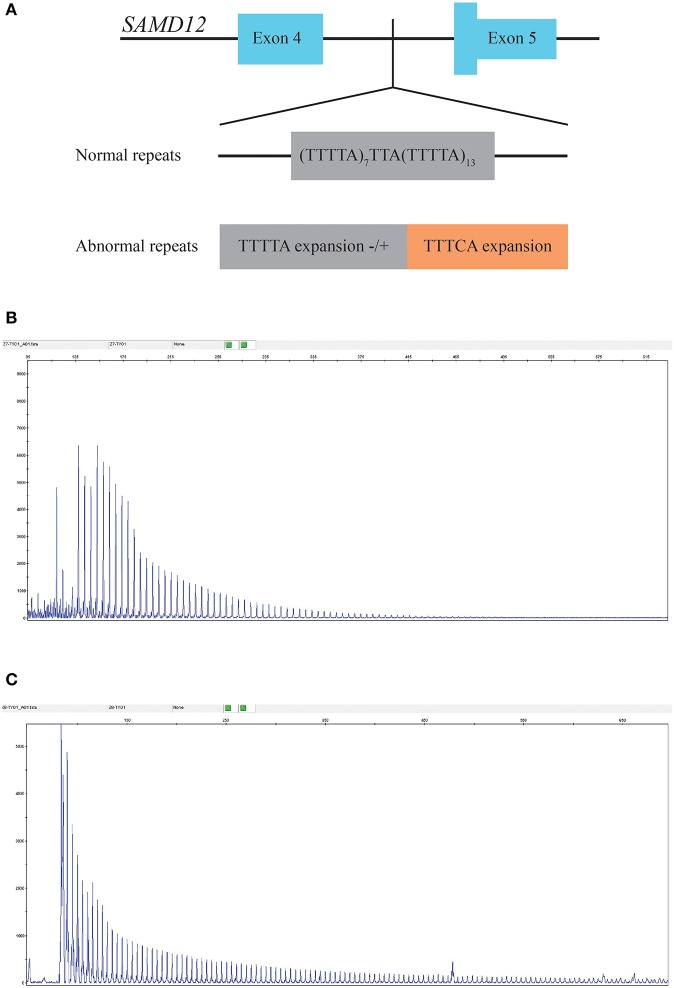
**(A)** Structure of the mutation in *SAMD12*. **(B)** RP-PCR indicated the existence of abnormal TTTTA expansion of III-7. **(C)** Repeat-primed PCR (RP-PCR) indicated the existence of abnormal TTTCA expansion of III-7.

Neither causal mutations cosegregated with the disease in the family nor any novel mutation was identified through WES (sequence depth of WES for individuals in the pedigree listed in Supplementary Figure 1), while an abnormal TTTCA expansion in *SAMD12* was identified by RP-PCR and then proved to be cosegregated in the pedigree. Minimum repeat size of TTTCA and TTTTA expansion for each patient estimated by RP-PCR are listed in Supplementary Table 2. Although TTTTA repeats were detected in all TTTCA-positive patients, there were still two unaffected individuals (III-21, IV-25) showing abnormal TTTTA expansions (Figure 3).

## Discussion

Since the first report from Japan in the 1990's, several pedigrees are reported worldwide and has been given various names including benign adult familial myoclonic epilepsy (BAFME), familial cortical myoclonic tremor with epilepsy (FCMTE), familial adult myoclonus epilepsy (FAME), and autosomal dominant cortical myoclonus and epilepsy (ADCME). BAFME is now known as a benign epilepsy syndrome with considerable clinical and genetic heterogeneity. van Rootselaar et al. summarized the characteristics and put forward diagnostic criteria of this disease (3). Subsequent researches further improved our understanding of this disorder. Our study offered the evidence of intronic pentanucleotide expansions in *SAMD12* from a new Chinese BAFME pedigree, which further confirmed the association between this expansion and the pathogenesis of BAFME.

In our pedigree, patients all met the diagnostic criteria proposed in 2005. Still, there were something special with their phenotype. First, there were only five (5/12) individuals presenting cortical tremor earlier than seizure, which was different from the conclusion that tremor was usually the first symptom (12). Besides, cognitive impairment was found by MMSE and VFT, which was basically consistent with our previous research (13). In addition, maternal clinical anticipation was observed in six mother/child pair. Since there was no alive father–child patient pair in this family, all were maternal, which varies from reports from another Chinese pedigree but consistent with that of Japanese (12, 14). No psychomotor development disorder or other disease such as night blindness reported in other BAFME pedigrees was witnessed. Our report further confirmed that BAFME was a non-progressing neurological disorder, but accompanied with cognitive impairment especially in language usage. Clinical anticipation did not occur in the severity of the disease but only in the age of onset. Larger samples are needed to confirm the cognitive impairment in the future.

Intronic pentanucleotide TTTCA and TTTTA repeat insertion of *SAMD12* gene were reported to be associated with BAFME recently (4). Our research in a new Chinese family confirmed again that the TTTCA abnormal expansion of *SAMD12* was the cause of BAFME, and the number of TTTCA expansion tended to differ from one to another, which showed instability of the expansions.

A recent report of BAFME pedigree identified interindividual instability of the pentanucleotide repeats and inversely correlated with age at onset of myoclonic tremor and seizure (11). Our study confirmed the interindividual instability, but minimum repeat size estimated by RP-PCR shows no relation to the age of onset in this BAFME family. Further studies such as Southern blot are needed to confirm the definite number of repeats. In addition, we also found that TTTCA insertions were accompanied by the abnormal TTTTA expansions in this Chinese pedigree, yet there were two unaffected individuals who showed abnormal TTTTA expansion, which indicated that TTTTA expansion may not be the cause of BAFME. Cen et al. found that some affected members had only 25–44 TTTTA repeats on the allele containing (TTTCA)n insertion, while in contrast, some unaffected individuals had alleles with 800 repeats in long-range PCR. They also observed an TTTTA pentanucleotide with more than 80 repeats in the healthy control, which was consistent with the report from Japan (4, 9). Our results further confirmed that TTTTA expansion might not be the cause of BAFME.

The mechanism how the expanded TTTCA and TTTTA repeats cause BAFME remains unclear up till now. However, similar repeat expansion has been proven to be the cause of many other neurodegenerative diseases such as fragile X syndrome (FXS) (15), several types of spinocerebellar ataxia (SCA31 and SCA37) (16, 17), *C9orf72-*related disorder (18), and, most recently, neuronal intranuclear inclusion disease (NIID) (19). These expansions are thought to cause disease through RNA-mediated toxicity. Repeated insertions expressed in brain and RNA lesions in the nucleus of the Purkinje cells were observed in SCA31 patient (20). While in FXS and *C9orf72*-related diseases, a loss of RNA and protein expression was observed and thought to be a result of hypermethylation and silencing of the promoter or nuclear RNA foci formation and haploid deficiency (21). According to previous studies, BAFME patients showed elevated choline/creatine ratio in the cerebellar cortex (22). The existence of cerebellar dysfunction was then proved in a Chinese BAFME pedigree along with altered cerebellar-cerebral functional connectivity (13, 23). In the report of the Japanese BAFME pedigrees, there were no obvious alterations in *SAMD12* transcript 1 levels observed in patient's brain, but RNA lesions were observed in cortical neurons and Purkinje cells, which was consistent with MRI findings, suggesting that RNA-mediated toxicity might be the basis of the pathogenesis in BAFME.

## Conclusion

In conclusion, BAFME is a non-progressing neurological disorder accompanied with cognitive impairment especially in language usage. Our study offered the evidence of intronic pentanucleotide expansions in *SAMD12* from a new Chinese BAFME pedigree, which further confirmed the association between this expansion and the pathogenesis of BAFME. The underlying mechanism of this disorder remains to be elucidated.

## Data Availability Statement

All datasets generated for this study are included in the article/Supplementary Material.

## Ethics Statement

The studies involving human participants were reviewed and approved by the Ethics Committee of Xiangya Hospital, Central South University. The patients/participants provided their written informed consent to participate in this study. Written informed consent was obtained from the individual(s) for the publication of any potentially identifiable images or data included in this article.

## Author Contributions

CL, LL, and BX: conception and design. CL, YS, XQ, WX, HL, SH, PZ, LL, and BX: collection and assembly of data. CL, YP, NP, RD, and WH: data analysis and interpretation. CL, YY, and LL: manuscript writing. All authors have approved the final manuscript.

### Conflict of Interest

The authors declare that the research was conducted in the absence of any commercial or financial relationships that could be construed as a potential conflict of interest.
