# Supplementary material for: TTTCA Repeat Expansion of SAMD12 in a New Benign Adult Familial Myoclonic Epilepsy Pedigree
Source: Front Neurol. 2020 Feb 26;11:68. doi: 10.3389/fneur.2020.00068 (PMC7055650; doi:10.3389/fneur.2020.00068)
Supplement: Supplementary file 1 [file Data_Sheet_1.pdf]

frontiers

Supplementary Materials

1 Supplementary Tables

Supplementary Table 1: Primers used for RP-PCR

Reaction	Primer name	Primers（5’→3’）
TTTTA repeat PCR	*SAMD12*STRF	CCTTATCCTTCTCTGAAATAATACTTCCAT
	*SAMD12*AAAATR	AAAATAAAATAAAATAAAATAAAATAAAAT
TTTCA repeat PCR	*SAMD12*TTTCAF	TTTCATTTCATTTCATTTCATTTCATT
	*SAMD*12FSTRR2	Gcctgggcaacaagagtga
Specific PCR	*SAMD12*PreF	tgtgcagCCATTGGTCCAGTCTT
	*SAMD12*PreR2	GCTGGCAAagttcagaggtcactt

Supplementary Table 2: Number of TTTTA and TTTCA repeats of the pedigree

Individuals	Number of TTTTA repeat	Number of TTTCA repeat	Individuals	Number of TTTTA repeat	Number of TTTCA repeat
III-7	>60	>111	III14	14	17
III-13	>60	>106	IV20	14	17
III-15	>57	>87	IV4	17	21
III-19	>58	>96	III6	17	20
III-20	>57	>100	III26	17	19
IV6	>57	>105	III23	17	20
IV15	>61	>103	IV12	17	21
IV19	>59	>100	IV14	17	21
IV23	>55	>105	III18	17	20
IV24	>58	>102	IV26	12	17
III-8	NA	NA	III30	17	19
IV-18	NA	NA	III22	12	18
III-21	>56	29	III32	19	20
IV-25	>58	17	IV3	17	20
IV1	17	20	III2	19	20
III4	20	21	IV28	17	20
IV22	17	19	IV21	17	20

NA, DNA not available.

Supplementary Table 3: Scales examination of BAFME patients and unrelated healthy controls

“/” represents data unavailable.

Individuals	sex	age	UMRS	FTRS	MMSE	DST	VFT
III-7	F	62	0	14	28	20	16
III-13	F	60	0	22	27	15	14
III-15	F	59	0	12	28	36	12
III-19	F	50	2	16	28	/	/
III-20	M	47	0	12	27	90	12
IV6	F	41	18	22	/	40	10
IV15	F	42	10	16	/	71	19
IV19	M	33	10	12	28	36	12
IV23	M	29	0	11	29	30	22
IV24	M	27	0	4	30	55	14
III-8	F	66	0	2	29	20	12
IV-18	F	30	0	/	/	/	/
C1	M	61	/	/	29	26	22
C2	F	44	/	/	30	29	11
C3	F	56	/	/	29	19	15
C4	F	27	/	/	30	72	24
C5	M	24			30	73	23
C6	F	27	/	/	30	90	22
C7	M	25	/	/	30	63	20
C8	M	38	/	/	30	67	17
C9	F	40	/	/	30	59	27
C10	M	53	/	/	29	27	20

2 Supplementary Figure

Supplementary Figure 1:

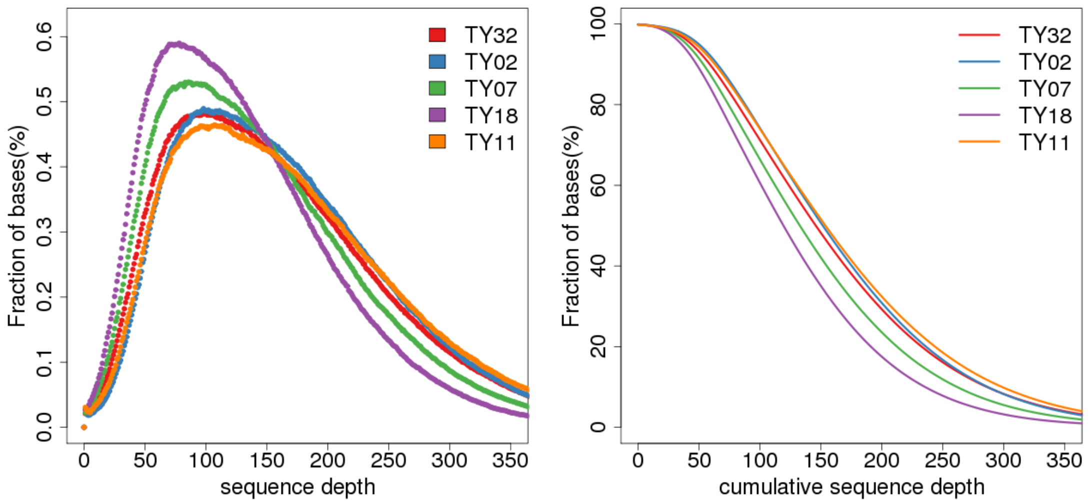

Figure 1: Sequence depth of WES for individuals in the pedigree
